# Insect Consumption to Address Undernutrition, a National Survey on the Prevalence of Insect Consumption among Adults and Vendors in Laos

**DOI:** 10.1371/journal.pone.0136458

**Published:** 2015-08-28

**Authors:** Hubert Barennes, Maniphet Phimmasane, Christian Rajaonarivo

**Affiliations:** 1 Institut de la Francophonie pour la Médecine Tropicale, Vientiane, Lao PDR; 2 Agence Nationale de Recherche sur le VIH et les Hépatites Phnom Penh, Cambodia; 3 ISPED, Centre INSERM U897-Epidemiologie-Biostatistique, Univ. Bordeaux, F-33000 Bordeaux, France; 4 Epidemiology Unit, Pasteur Institute, Phnom Penh, Cambodia; Hunter College, UNITED STATES

## Abstract

**Background:**

Insect consumption (entomophagy) is a potentially high nutritious and healthy source of food with high fat, protein, vitamin, fiber and micronutrient content. At least 2 billion people globally eat insects (over 1900 edible species) though this habit is regarded negatively by others. There is a limited amount of data on the perception and consumption of insects. We conducted a national cross-sectional survey in the Lao People’s Democratic Republic (Laos) to assess the prevalence and characteristics of insect consumption among adult lay people and insect vendors.

**Methods:**

We conducted a multi stage randomized national survey in 1303 households in 96 villages in 16 Lao provinces. Three insect vendors or collectors per village were also included. A standardized pretested questionnaire addressed the following issues: socioeconomic characteristics, type of insects consumed and frequency of consumption, reasons and trends in consumption as well as reports on side effects, over the last 10 years.

**Results:**

A total of 1059 adults (Sex ratio F/M: 1.2, 30 ethnic groups), and 256 vendors were enrolled. A total of 1025 (96.8%) lay people were currently insect consumers, 135 (13.0%) daily or weekly consumers, and 322 (31.1%) consumed several times per month. For the majority (575, 55.6%) the consumption was infrequent (less than a few times per year) and only 22 (2%) had never eaten insects. Consumption started in childhood. Insect availability was seasonal (670, 63.2%) and respondents would have eaten more insects, if they had been more available (919, 86.7%). Hmong and Leu ethnic groups had significantly lower consumption levels than the general population. Eggs of weaver ants, short-tailed crickets, crickets, grasshoppers, and cicadas were the top 5 insects consumed. Consumption had decreased in the last decade, mostly due to less availability (869; 84.0%) and change of life (29; 5.5%). Of 1059, 80 (7.5%) reported allergy problems and 106 (10.0%) reported some use in traditional medicine. A total of 874 (82.6%) were regular collectors.

Insect vendors (Sex ratio F/M: 5.3) were also collectors (185; 72.2%). They dedicated a mean time of 4.7 hours during the last harvesting period. The majority sold insects at markets (141, 55.0%). They had earned, on average, USD 6.0 the day before. Five insects (weaver ant eggs; bamboo worms; short-tailed crickets; crickets; wasps) represented 85% of the market.

**Conclusion:**

Entomophagy is general in Laos, and well accepted despite a decreasing trend in consumption over the last decade. Its contribution to the Lao diet is limited to a minority of frequent consumers. Income through insect sales benefits mostly women. Consumption varies according to ethnicity, residence and season. Development of insect farming is still at an early stage. It could however increase availability of insects and contribute to the generation of income.

## Introduction

Worldwide an estimated 805 million people are malnourished, with a total food energy deficit of 67.6 billion kcal/day (84 kcal/day/person) [1]. Insects have high nutritive values and represent a potentially healthy source of food with high fat, protein (13–77% of dry matter) vitamin, fibre and mineral content[2]. They are easy to breed and harvest. They have a high fecundity, can produce many broods per year, present high feed conversion efficiency, have low space requirement, and are omnivorous. Insects can contribute to world food security and act as an alternative food source, especially for meat production and fish meal [2,3].

At least 2 billion people globally eat insects in over 113 entomophageous countries though this habit is regarded negatively or as revolting by others [4–6]. More than 1900 species are consumed by local populations globally but insect consumption (entomophagy) shows an unequal distribution. The most common edible insect groups are beetles (Coleoptera), caterpillars (Lepidoptera) and bees, wasps and ants (Hymenoptera), grasshoppers, locusts and crickets (Orthoptera), cicadas, leafhoppers, planthoppers, scale insects and true bugs (Hemiptera), termites (Isoptera), dragonflies (Odonata) and flies (Diptera). Many people eat insects out of choice, largely because of the palatability of the insects and their established place in local food cultures [1,5].

The nutritional values of edible insects is highly variable because of the wide range of edible insect species [7]. This also varies depending on the metamorphic stage of the insect, their habitat and diet as well as preparation and processing methods (e.g. dried, boiled or fried) and storage before consumption. Despite these significant variations, many edible insects provide satisfactory amounts of energy and proteins that meet amino acid requirements for humans, are high in mono-unsaturated and/or poly-unsaturated fatty acids (including the essential linoleic and α-linolenic acids), and are rich in micronutrients such as copper, iron, magnesium, manganese, phosphorous, selenium and zinc[8], as well as riboflavin, pantothenic acid, biotin and, in some cases, folic acid [3].

We provide a few examples of the potential use of insects for human nutrition. According to the FAO the composition of unsaturated omega-3 and six fatty acids in mealworms is comparable with that of fish and higher than in cattle and pigs. Its protein, vitamin and mineral content are similar to that in fish and meat [5]. Insects that contain amino acids such as lysine, missing in some cereals or vegetable, are of particular interest to people having cereals (maize, rice) or cassava as key staples. Insects, particularly terrestrial ones, which are rich in polyunsaturated fatty acids could provide these essential fatty acids to local diets particularly in landlocked, developing countries such as Laos with lower access to fish food sources [7]. Insects containing vitamin B1 could be beneficial in Southeast Asian countries where thiamine deficiency in breastfeeding mothers remains the cause of high infant mortality or where sublevels of thiamine have been reported [9–12]. Insects could provide easy protein inputs in areas where people are reluctant to eat or have limited access to more common protein sources such as pork, beef, poultry, milk and eggs[13]. Wasps that have the highest protein content [4] are largely consumed by Chinese people [14].

Last but not least, the production of food from insects can be obtained at low cost [1,5]. On average, “insects can convert 2 kg of feed into 1 kg of insect mass, whereas cattle require 8 kg of feed to produce 1 kg of body weight gain”. Increasing the world supply of animal protein by 10% through mass production of insects, is estimated sufficient to challenge malnutrition problems and to decrease the pressure of other protein sources [1,15].

Many Southeast Asians have a long tradition of eating insects [4,13,16] and at local Asian markets, insects are objects of curiosity for tourists. Over 164 different edible species have been reported in Lao, Thailand and Myanmar [17].

Laos is a low income, traditional, multi-ethnic and multi-lingual country in Southeast Asia. Despite dramatic economic improvements over the last decades, stunting is highly prevalent nationally (44%) among children under 5 years old [18]. Wasting is much less prevalent (about 6%), but some clusters of high prevalence of wasting have been described in the southern provinces [19]. Inappropriate nutritional practices such as postpartum food taboos [9,19], unhealthy feeding practices, breastfeeding misconceptions or formula misuse [20,21], insufficient global and nutrients intakes are responsible for severe micronutrient deficiency and child progressive growth deterioration [22–25]. As an example, clinical and non clinically apparent thiamine deficiency have been reported among infants, children and adults [10–12,26] and is responsible for sudden infant deaths [10]. The level of undernutrition in Lao children has led to various initiatives [22,24,25,27–29] including the research of locally neglected sources of nutrients which inspired this work.

Lao people have also a long tradition of collecting and eating insects [17]. Rural folk eat insects as their main dish, whereas urban people eat them either as a main dish or snack, or both. A previous survey in Vientiane province described a total of 21 species of edible insects collected by the villagers in a forest village and its related market [30]. The nutritional values of Lao insects that can be estimated from neighbouring Thailand data, suggests that they have a nutritional potential [4]. The amino acid score of silkworm pupae reaches 100, followed by bamboo caterpillars (77.5), house crickets (68.7), wasps (59.4), Bombay locusts (55.8) and scarab beetles (34.2). Insects that have an optimal ratio of fatty acid are house crickets, short-tailed crickets, Bombay locusts and scarab beetles[4].

However, these results may not represent the community practice of entomophagy in a multi-ethnic country. Additional information is needed regarding the nationwide habits and practices of insect collection and consumption before the implementation of nutritional programs based on insect consumption. We conducted a national cross-sectional survey to assess the prevalence and characteristics of insect consumption among adult population during a national multi-stage, random sampling in the Lao People’s Democratic Republic (Laos).

## Methods

### Study site

Laos is one of the poorest countries in Southeast Asia, and ranked 139 out of 187 countries evaluated according to the Human Development Index [5,31]. After years of being a landlocked country, surrounded by Vietnam, Cambodia, Thailand, China, and Myanmar, it is rapidly becoming a land-linked country with the opening of roads and borders following the liberalization of the economy in 1986. The per capita income was USD 1010 in 2009 at the time of the survey [32]. About 80% of the Lao population work in the informal sector, primarily in subsistence agriculture and 44% of the population live below the international poverty line of USD 1.25 per day.

### Sampling method

**Fig 1:**
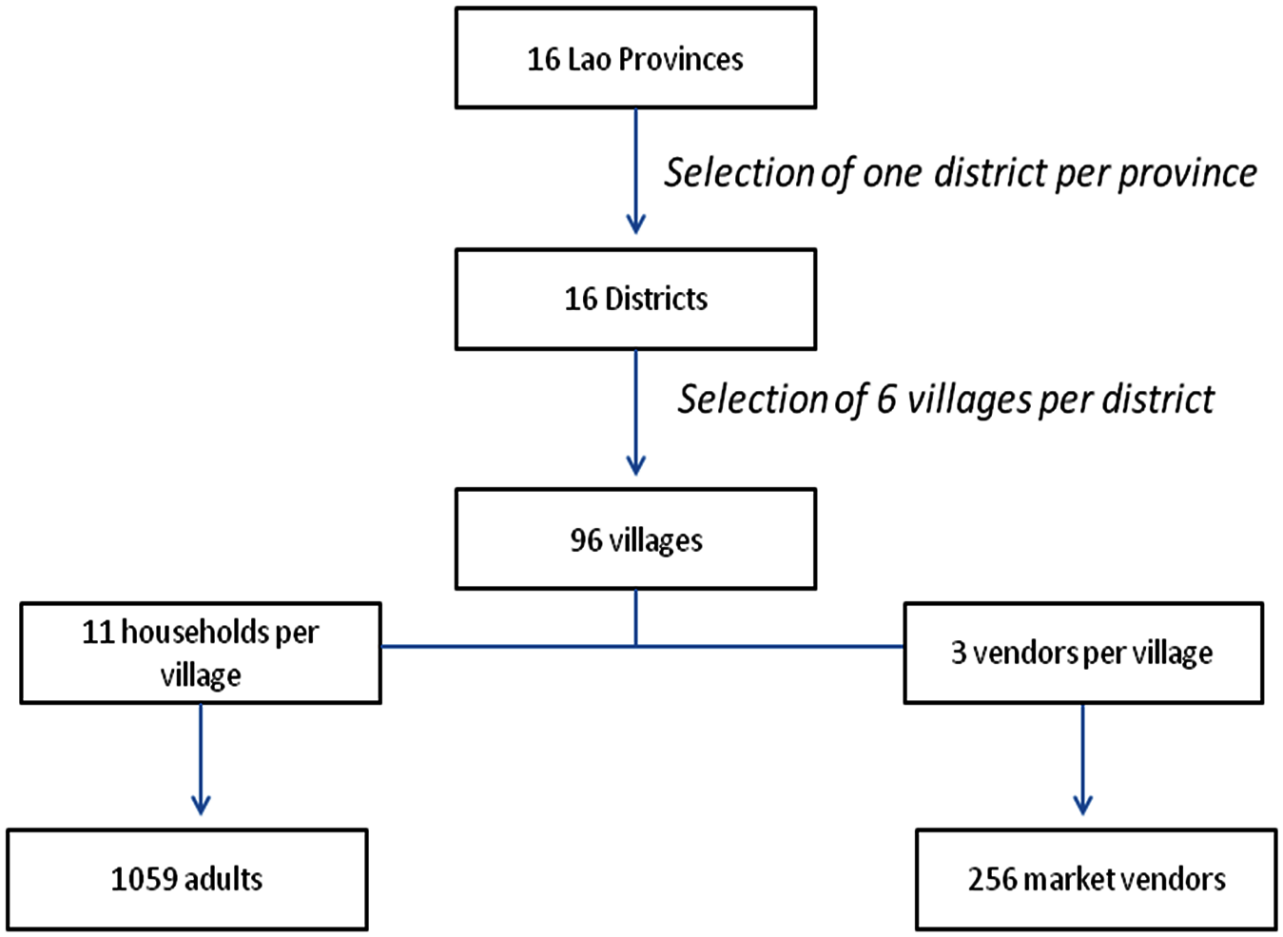
Flow chart of the national edible insect survey in Laos.

We conducted a cross-sectional survey in 16 of 17 provinces outside Vientiane Capital in March 2010. The flow chart of the survey is presented Fig 1.

Briefly, in each province one district was randomly selected using a random numbers list. A multi-stage random sampling was applied within each selected district. In the first stage, 6 villages were randomly selected from the 2005 census list of all villages in each selected district[33]. In the second stage, 11 households were randomly selected from the list of households in every village. If a selected household was not available, the investigating team returned later. In case of no success the house next door was chosen complying with inclusion criteria and number.

In addition 3 vendors or collectors of insects per village were included. If no vendors or collectors were available in the villages, teams were asked to stop whenever they came across a market or heard of potential vendors/collectors in a village.

### Inclusion criteria

In each selected household, one available adult was randomly included and interviewed in Lao language. If multiple adults were eligible in the same household a randomization list, compiled prior to the survey, was used. This list was also used to avoid unbalanced gender and age ratios. Reasons for refusal of participation were recorded.

### Data collection

Data collection was done by 8 teams (teams of 2 physicians and one local attendant or facilitator as appropriate). Investigators were medical physicians attending a master’s course with special lectures in epidemiology, field research and public health at the “Institute pour la Francophonie de Medecine Tropicale” (IFMT). They received specific training and conducted three pre-testing investigations in Vientiane markets and surrounding villages from January to March 2010. They were assisted by 6 supervisors and a team leader. Among them, some could speak other Lao ethnic languages such as Khmu (1) and Hmong (3).

A 56-main item structured questionnaire was used. The questionnaire addressed the following issues: socioeconomic characteristics of interviewees, name of insects consumed, reasons for consumption, frequency of consumption by day, week or months, and trends in consumption in the past 10 years, names of insects collected and sold, reports of side effects and toxicity. Questions were also asked regarding the time they had spent collecting insects during the last collection. For those who bought insects, prices paid at the last purchase. The quantity of insects consumed or collected was not assessed. Due to the large variety of insects presented it was thought non-realistic, in the context of this community survey, to assess the quantity with reliable measurements. We also asked if any other insects were eaten and if the answer was in the affirmative, then the names of the insects consumed. A few questions on beliefs, and traditional uses of insects were open questions.

In addition, teams used a poster with 26 pictures of available edible insect species. These were collected during the pretesting of the questionnaire in the markets and during a review of the literature. This poster included 4 dummy images (frog, snake, crab, and scorpion). These 4 images were included in order to check if people used the same term for the insect in different languages and if they understood clearly what the insects were.

### Data entry and analysis

Data was entered in EpiData freeware (S1 Dataset). All records were cross-checked with the original data sheets. Analyses were carried out using STATA, Version 8 (Stata Corporation, College Station, TX, USA). Data is presented as number and frequency (%), mean and 95% Confidence interval (95% CI).

Chi Square and Fisher’s exact tests, Students’t-test and Wilcoxon test were used to compare categorical variables and continuous data, respectively. P<0.05 was considered as statistically significant.

### Sample size and sample calculation

The sample size was calculated using Stata by estimating the prevalence of insect consumption (60%), a 5% precision, 90% power, and 5% of anticipation for drop-out or refusal. The total minimum required sample size was 988 but was rounded off to 1050 households. A total of 30 vendors per team was considered a feasible target.

### Ethics

This study formed part of a master’s course at “Institut Francophone pours la Médecine Tropicale” (IFMT, Vientiane, Laos). All study participants were informed about the study in Lao language and provided with an information sheet describing the study. For non-Lao speakers, translation was performed by a native speaker into their own language. People were included in the study only after they gave written informed consent. For those who were illiterate, oral consent in the presence of a witness, was obtained. Agreement was recorded on the consent form. Ethical clearance for the study was sought and the procedure of the whole study was approved by the Lao Medical Ethics Committee. In addition, the study was conducted with the agreement of the Lao health authorities.

## Results

### Characteristics of study population

The population covered by the survey represents an estimated total of 6806 people and 1303 households in 96 randomised villages in 16 provinces and districts (Fig 1). Few refusals to participate in the survey were observed among the population. Reasons for refusal were unrelated to the survey topic: lack of time, busy, unknown reasons. One selected village had to be changed due to the refusal of the village headman. A total of 1059 adults (Table 1) and 256 vendors were available for analyses. Some questions were not unanswered by a few respondents; hence the number of respondents varied from 1032 to 1059 and is indicated on the tables.

**Table 1:** Characteristics of population during national edible insect survey in Laos.

	Interviewees	
	n = 1059	%
Age (years) *	43.3	(42–44)
Sex ratio (F/M)	1.2 (592/467)	
Animist	354	33.4
Buddhist	677	63.9
Primary education	445	42.0
Illiterate	276	26.0
Main occupation		
-Farmer	667	62.9
-Household	134	12.6
-Business	83	7.8
-Civil servant	70	6.6
-Student	41	3.8
-Worker	23	2.1
-Retired	21	1.9
Housing		
No electricity	222	20.9
No tap water	596	56.2
No latrines	191	18.0
Community latrines	153	14.4
No motorbike	160	15.1
No refrigerator	432	40.7
No radio	284	26.8
No TV	120	11.3
Mean monthly income (n = 994) **	93.6 (88.8–110.3)	

Mean and 95% confidence interval,

** US dollars: 1 $ = 8000 kip.

Overall, respondents were middle aged (43.3 years), had primary education (42%), were mostly farmers (62.9%), lived in a house without electricity (20.9%), had no tap water (56.2%) or no latrines (18%).

Their self-reported monthly income was USD 93.6 (95% CI: 92.3–93.6).

The characteristics of interviewees were in agreement with the 2005 Lao national census (Table 1)[33]. Lay people belonged to 30 ethnic groups with 10 ethnic groups accounting for 92.2% of participants (Table 2).

**Table 2:** Main ethnic groups represented in the edible survey in Laos*.

	Interviewees	
	n = 1059	%
Lao	543	51.3
Khamu	155	14.6
Hmong	118	11.1
Leu	56	5.2
Phounoy	24	2.2
Others	20	1.8
Kui	15	1.4
Lavy	13	1.2
Alack	11	1.0
Oey	11	1.0
Kor	10	0.9

*Only ethnic group represented by more than 10 people.

### Characteristics of insect consumption

The most popular insects are shown in Table 3. Perceptions of insects’ nutritive value are shown in Table 4. Main practices regarding insect consumption and the trend of consumption over the last decade in Table 5.

**Table 3:** Frequency of insect consumption of the most common insects during the national edible insect survey.

Insect name (Lao name)	intervieweesn = 1059	%
Weaver ant eggs (Khai Mot deng)	336	18.1
Short-tailed Cricket (Chi nai)	309	16.7
Cricket (Chi lor)	232	12.5
Grasshopper (Tak tene)	156	8.4
Cicada (Chak chan)	141	7.6
Bamboo worm (To mir, Douangnormai)	121	6.5
Wasp (Tor)	89	4.8
Mole Cricket (Meing xone)	76	4.1
Dragonfly (Meingnaagam)	64	3.4
Dung beetle (Meingchudchii)	33	1.7
Long-legged katidyd(Chong Cho))	25	1.3
Dung beetle (Meingchudchii)	18	0.9
Giant water bug (Meing da)	15	0.8
Diving beetle (Meing TabTao)	14	0.7
Snout beetle (Meing nor mai)	14	0.7
Stink bug 2 (Meing Khieng)	11	0.6

(Lao names of insects)

Only response over 10 insects are represented.

**Table 4:** Perception of edible insect nutritive value.

	n = 1033	%	95% CI
What kind of meal are insects?			
a complete meal	845	81.8	79.3–84.1
a snack	188	18.1	15.9–20.7
Both	373	36.1	33.2–39.1
Does not know	486	47	42.9–49.0
Good nutritive content	624	60.4	55.8–61.9
Reports a nutritional value	571	55.2	50.9–57.0
-Vitamin	207	20	17.6–22.6
-Fat	140	13.5	11.5–15.7
-Same as meat	98	9.4	7.7–11.4
-Sugar (sweet)	62	6	4.6–7.6
-Calcium	54	5.2	3.9–6.7

**Table 5:** Main practices and current trends of insect consumption in Laos.

	Respondents		
	n = 1059	(%)	95%CI
Currently eats insects	1024	96.6	95.4–97.6
Frequency of consumption	n = 1033		
-Very frequent (Daily or several times/a week)	135	13	11.0–15.2
-Frequent (several times a month)	322	31.1	28.3–34.1
-Occasional (Several times a year)	304	29.4	26.6–32.3
-Rare	271	26.2	23.5–29.0
Where/On what occasion are you eating insects?			
-with the family	845	81.8	79.3–84.1
-with friends	110	10.6	8.4–12.7
-during feast	40	3.8	2.7–5.2
Compared to previous 10 years consumption is	n = 1033	(%)	
-Less consumption	526	50.9	47.8–54.0
-Same consumption	372	36	33.0–39.0
-More consumption	126	12.1	10.2–14.3
-Do not know	11	1.0	0.5–1.8
Reason of decrease of consumption	N = 525	(%)	
- Insects less available	441	84.0	441
- Standard of life*	29	5.5	3.7–7.8
- Insect became more expensive than before	14	2.6	1.4–4.4
- I am too old to eat	11	2.1	1.0–3.7
- We have something else to eat	8	1.5	0.6–2.9
- Others	20	4.1	2.3–5.8
Availability depends on season	670	63.2	60.2–66.1
-Not available all year long	119	11.2	9.3–13.2
-Will eat more if available	919	86.7	84.5–88.7
Started eating as a child	1.001	94.4	92.9–95.8
All family members eat	976	92.3	90.3–93.7
Eat insect eggs	870	82.1	79.7–84.4
Acceptability			
-Good advantages to eat insects*	885	83.5	81.1–85.7
-No problems with insect	830	78.3	19.0–24.1
Ever feel itchy after eating	81	7.7	6.1–9.4
Children ever sick after eating	52	4.9	3.6–6.3
Usual collector of insect	874	82.5	80.1–84.7
-Duration at last collect (hours)**	874	3.0	2.8–3.1
**Ever buy insects?** *** **(n = 1032)**	**371**	**34.9**	33.0–38.9
-Once a year	154	14.9	12.8–17.2
-Every month	168	16.2	13.7–18.2
-Less than 4 times a month	29	2.8	1.8–4.0
-Several time a week	17	1.6	0.9–2.6
**Last spending on insects (USD) (n = 367)** ***	1.28		1.1–1.3

1 US dollar = 8000 kip

Mean, and 95% confidence interval (95%CI).

*The main reasons of insect’s popularity were mostly their taste (603, 68.2%), their easiness to collect, and the potential income that can be generated when collected in great numbers.

** 629 (71.9%) spent less than 3 hours collecting insects, 157 (17.9%) between 3 and 6 hours, and 88 (10%) more than 6 hours.

*** The median expense at last purchase varied from 0.6 US dollars in Phounoy ethnic group up to 1.5 in Hmong groups (p = 0.003). 154 (14.5%) reported to buy once a year. The specific reason for this rare event was not assessed.

Eggs of weaver ants, short-tailed crickets, crickets, grasshoppers and cicadas were the top 5 insects consumed (Table 3). The majority of insects (623; 58.8%) were consumed on the same day, or for later consumption (146; 13.7%) or both (263; 24.8%). Villagers reported another 20 edible insects whose names were only known in the local dialect. This leads to a provisional number of about 46 commonly eaten insects in Laos. Among the 5 main ethnic groups that could be analysed separately (more than 20 people included) Hmong and Leu consumed mostly bamboo worms, wasps and giant water bugs and had lower consumption levels than the general population (85% and 93% versus 97% respectively) (P<0.001).

Consumption of other types of insect eggs was anecdotic: wasps (18, 2.07), bamboo worms (16, 1.84), and snout beetles (10, 1.15). Consumption of insect eggs was higher among Lao Loum (94%) and Khmu (95%) than in the other ethnic groups (P<0.001).

The majority of interviewees (81.8%) considered insects a staple food while the rest of interviewees (193; 18.6%) considered them a complementary food or snack (Table 4). Half the people (571, 55.2%) had some notion of the nutritive value of insects.

### Prevalence of insect consumption in Laos

**Fig 2:**
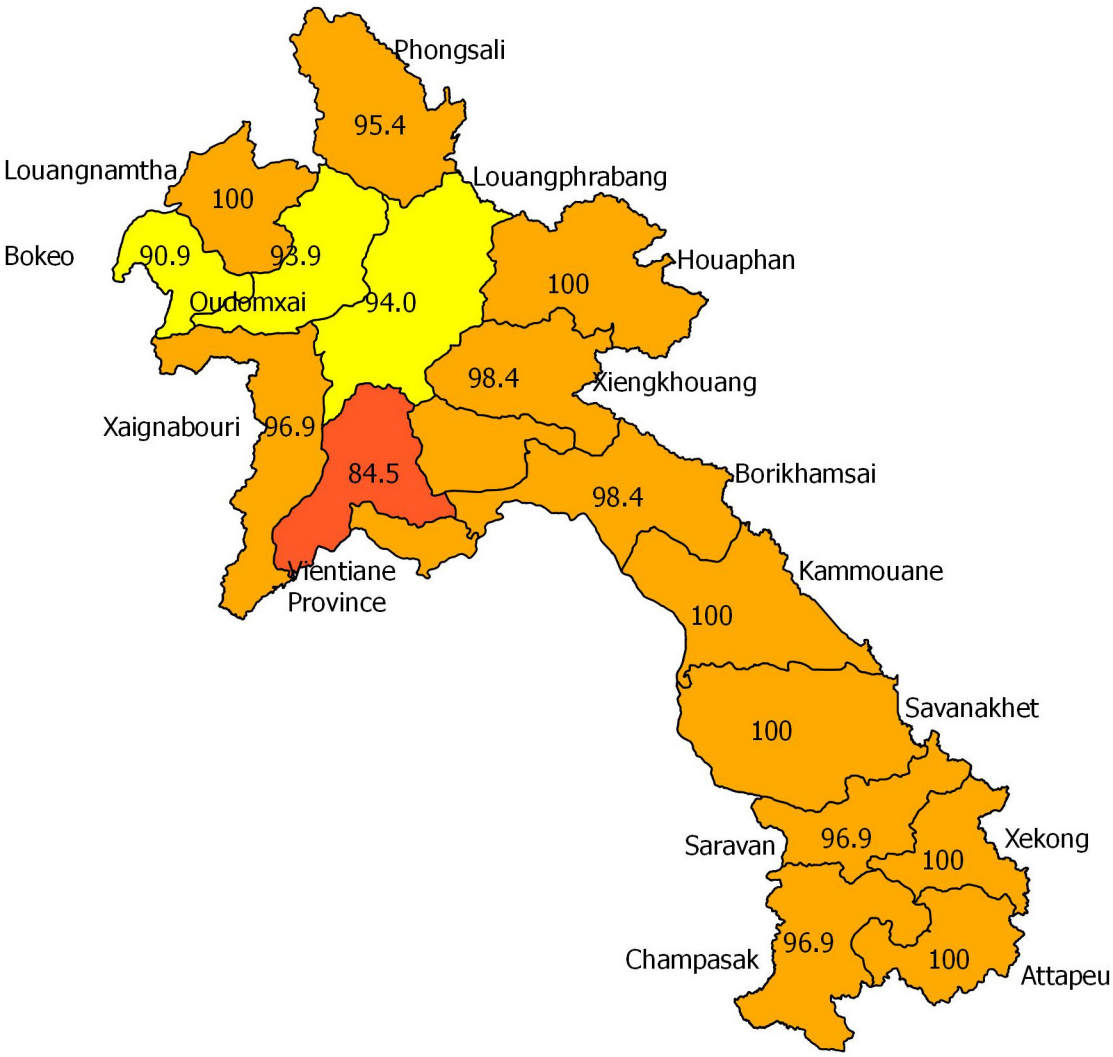
Geographical distribution of insect consumers in Laos.

The prevalence of insect consumption in the population was above 95% in all but two provinces (Bokeo/Hueasay and Vientiane/Fuang; 90% and 85%, respectively) (Fig 2). Of 1059 interviewees, 1024 (96.6%) were current insect consumers and only 22 (2%) had never eaten insects (Table 5). Insect consumption was well accepted and the majority found it advantageous to eat insects (885, 83.5%) or did not see a problem in eating insects. The main reasons for insects popularity were mostly related to their taste (603, 68.2%), their easiness to collect (86, and the potential income generated from collecting insects in great numbers.

For the majority (575, 55.6%) consumption was infrequent (less than a few times per year). About 135 (13.0%) reported a weekly or daily consumption. The quantity of insects consumed by those frequent consumers was not assessed. Insects were mostly eaten with the family (845, 81.8%), with friends (110, 10.6%) and less frequently during feasts (40, 3.8%).

A total of 526 (50.9%) reported a decreased consumption over the last decade (Table 5). The main reason was due to a reduced availability of insects (441, 84.0%), change of life standard (29, 5.5%) or insect’s costs (14, 2.6%). For a few families in Southern Laos, insects are particularly valued during times of food shortages. Families reported that it was the only food available during such times.

Availability was seasonal (670, 63.2%) and nearly all respondents would have eaten more if insects were available (919, 86.7%) or if insects were less expensive.

Insect consumption began in childhood (94.5%) and generally the whole family were insect consumers with the exception of a few (81; 7.7%) who experienced allergies (81; 7.7%). Problems were mostly with grasshoppers or stink bugs (38; 46.9%). Other health problems were rarely reported (less than 10 responses).

A total of 874 (82.5%) were regular collectors and spent an average of 3 hours the last time they had collected insects (Table 5). Typically crickets were collected from March to December, stink bugs from February to May and cicadas from March to May; grasshoppers were collected all year around.

Table 5 further shows that the majority of interviewees (662, 64.1%) never bought insects. Among 370 responders (34.9%) who had the habit of buying insects, only 46 (12.4% of buyers) did it on a daily or weekly basis, 168 (45.4%) on a monthly basis and 154 (41.6%) less frequently (Table 5). Lao Loum were the only group with a high proportion of insect buyers (57%) (p<0.001).

Wasps are usually considered the most valuable insects among consumers. Insects were served in different ways: sold by unit or by plates; fried, smoked, steamed, toasted with spices or prepared on skewers. The average price of insects was variable (from USD 0.13 for a plate of bush crickets, up to USD 2.6 for wasps). The average expense at the last purchase was USD1.2 (95% CI: 1.1–1.3). A small group of frequent buyers had spent on average USD 12 the previous time.

### Characteristics and practices of insect’s vendors in Laos

Vendors’ characteristics are shown in Table 6. They were mostly women (Sex ratio F/M: 5.3). They belonged to 22 ethnic groups and 83 (32.6%) were illiterate. They had already spent a mean of 6.9 years as vendors. This activity was a full time position for 49 (19.1%) who sold insects exclusively, or part-time (more than twice a week) for the majority 164 (64.0%). The majority of vendors (204, 80.3%) also sold other products such as food, fruit and vegetables. 185 (72.2%) were also collectors and dedicated a mean time of 4.7 hours (95% CI: 4.4–4.9) during the last harvesting period. Others received their insects from trappers (62; 24.2%) but rarely from insect farms. They had earned on average USD 6.0 the day before the survey. Five insects (weaver ant eggs; bamboo worms; short-tailed crickets; crickets; wasps) represented 85% of the market. The main customers were villagers (210, 82.6), strangers (87; 34.3%), markets (43, 16.9%), and restaurants (20, 7.8%). According to vendors, taste (187, 73.6%) and eating habit (115, 45.2%) were the two major reasons for people buying insects. Being a delicacy and readily available were also reported as minor reasons (41, 16.2%).

**Table 6:** Main characteristics of insect vendors.

	Vendors	
	n = 254	%
Age (years) *	37.3	36–39
Sex (F)	214	84.2
Illiterate	83	32.6
Years in the profession *	6.9	6.1–7.6
-Daily vendor/collector	90	35.5
-Weekly vendors/collector	73	28.8
Source of insects		
-Harvest insect	185	72.3
-Wholesaler or collectors	62	24.2
-Insect farms	7	2.7
Mean time to collect insects/day (hours)**	4.7	4.4–4.9
Total income the day before the survey ***	5.8	5.7–6.7
General consumer expenses (US dollars)***	2.3	1.9–2.8

* Mean and 95% confidence interval,

** at last collect of insects,

*** US dollars: 1 $ = 8000 kip.

The seasonal and geographical availability of insects was particularly evident for vendors. Due to low harvest during the period of the survey in northern region, very few vendors were present in the northern markets which differed from the southern and central provinces.

## Discussion

This is the first national survey to report on the consumption of edible insects with a fair representativeness in Laos, evidenced by the general characteristics of the study population. The results show that insect consumption is a widespread family practice in all the Lao ethnic groups, including both urban and rural areas. As suggested by Yhoung-aree in 1997, edible insects should no longer be considered unconventional[13]. However, despite being very popular, daily or weekly consumers represent only a minority of the consumers. Consumption is rather occasional and has decreased over the last decade due to the change of living standards and a decrease in the availability of insects. A vast majority of Lao people still practice familial insect harvesting and only a small part of the population buy insects. These results suggest further evaluation of the nutritional impact of insect consumption among frequent and less frequent consumers. It also suggests documenting how to extend the insect availability and consumption.

The interviewees reported a decrease in consumption over the past decade, mostly due to a decreased and seasonal insect availability but were ready to eat more insect if the problem of insect availability was solved. Furthermore, insect farms were not a frequent source of insects for the population. Only 7 insect farms were reported by interviewees. The majority of the population reported insect harvesting practice and rather infrequent practices of insects purchase. This suggests that insect farming is probably at an early stage in Laos, unlike in Thailand.

Our survey suggests that there are opportunities and economic incentives to developing insect farming in Laos.

Decreasing trends in consumption of insects in the last decade has been reported by consumers but not by vendors, which shows that selling of insects is currently an active and productive market in Laos. Similar observations were made among vendors at a Lao market in a previous survey in Vientiane province [30]. In this survey, interviewees stated that they were spending increasingly more time gathering comparable quantities of edible insects compared to ten years earlier due to a larger number of insect collectors competing for the insect stock.

Insects represented an interesting additional source of income, mostly for women, bearing in mind that 44% of the Lao population lives below the international poverty line of USD1.25.

People and vendors also reported the seasonal non availability of insects which could be avoided by insect farming.

Similar optimization of small scale production has been successful in Cambodia and Thailand [9,16]. Farming may help to render insect farming from a seasonal activity to a year round activity and may be a source of additional income for families in remote areas. In Thailand selling insects represents a significant improvement in the economic status of poor farmers [4]. Between 2011 and 2013 FAO developed a project to enhance insect farming and commercialization in Laos.

Lao is severely affected by stunting, malnutrition, and micro nutrients deficiencies [18,27,34,35]. For example, it has been shown that in Lao adults, breastfeeding mothers, and as a result infants, were chronically deficient in thiamine [10,36,37]. Most of the nutrition interventions in Laos rely on supplementary and external components such as multivitamin sprinkles [38]. Promoting locally available food is seldom advocated. The majority of interviewees considered insects a “complete meal” and others more as a snack. Some families from the south stated that they collected and ate insects during food shortages. These results support the role of insects during times of food insecurities and the fragile balance of it in Laos. Indeed, insects are particularly rich in protein, as well as essential fatty acids, vitamins, including thiamine and some micronutrients and should be considered as part of the global strategy to fight undernutrition[1].

However, due to the disparity of nutritive values of insects which is dependent on the species as well as feeding and cooking procedures, it would be interesting to locally evaluate the real nutritional values of insects according to these criteria as was done in Thailand [3,4]. Using insects to fight undernutrition also supports development of insect farming which could help with standard procedures to maintain sufficient nutritive levels.

This study was not designed to perform an inventory of edible insects in Laos, but rather to assess the prevalence rate of consumption and practices. Villagers reported 20 more edible insects which were only identified by their Lao names. This suggests that around 46 edibles insect are commonly consumed in Laos, a number close to that of Thailand but higher than previously described in Laos, and probably deserves further description [5][20]. Finally, as for other non-wood forest products, deforestation in Laos poses a serious threat to insect habitats and availability. This is a concern for those who benefit most from them i.e. poor, women and children and some ethnic groups. This issue was raised by a few groups. They were ready to protect insects in order for their children to also be able to consume them in the future. Similarly, promoting insect consumption may be an interesting way to protect their source i.e. the forests and increase the awareness of protecting this valuable source of food for the Lao population. Due to the threat on the sustainability of edible forest product resources, developing insect farming could provide a solution to maintaining the availability of edible insects in some regions of Laos. This will help maintain a traditional diet in the population.

The majority of vendors were female, illiterate and reported a median raw income of USD 5–6 dollars per day, a little less than reported previously [17]. Despite its seasonality in the northern part of Laos, the insect business can be seen positively as a means to reinforce women’s ability to own a business and become financially independent.

A few points about insects have to be further discussed such as the side effects of insect consumption: allergies to insects and unsafe storage of insects. A few consumers (81, 7.6%) reported allergy problems after eating insects, mostly grasshoppers and stink bugs. The rate was low and none reported severe anaphylaxis but interviewees clearly denounced the danger from wasps and a few others insects. Allergies to insects were rare but could be highly fatal [39]. Hymenoptera including bees, wasps, hornets, yellow jackets, and ants, are responsible for the majority of the fatal and near-fatal sting events. Screening people with potential allergies and recommending insect-avoidance or insect-contact strategies might be the only options, together with providing immediate epinephrine in a country where more sophisticated venom specific immunoglobulin is not available[39].

Most of the insects were harvested for immediate consumption, the others for later consumption. Potential contamination of insects sold at the market without proper storage might be an issue that needs to be addressed [3]. Rapid spoilage of raw edible insects is a limitation and decontamination methods and storage conditions have to be evaluated and developed. Aflatoxin, parasitoses, food poisoning and potential microbial threat have been reported elsewhere and safety of the insects sold should be addressed while implementing edible insect programs[3].

### Survey limitations

This survey has several limitations. Some limitations are related to usual anamnesis and recall bias and the difficulty to quantify the amount of insect consumption. The interview set at home could explain a slightly skewed sex ratio. To avoid this, investigators had a randomized list prepared before the survey to allow gender and age equity among interviewees. The necessary use of translators in some ethnic groups may have hampered the quality of some responses. The March-April season (hot season) is a season where some species are not available and it had an impact on the vendor profiles. Finally, this survey did not address the total insect nutrient intake in the population. It was not possible to know how much the consumption of edible insects represents the daily diet of the population. More information is also needed regarding the impact of pesticides and insect consumption in Laos. The survey also did not detail the insect trade chain or the way to harvest insects. These points probably deserve further exploration.

## Conclusion

This first national survey of edible insects in Laos shows that insect consumption remains popular, and well accepted despite a decreasing trend in consumption in the last decade. Its contribution to the Lao diet is limited to a minority of frequent consumers. The availability of insects varies according to season and geographic location, and pattern of consumption differs lightly according to ethnicity. The population is ready to eat insects more frequently if the two problems of seasonal availability and cost are solved. Most of the Lao population harvest their own insects and only a third of them are regular buyers. The sale of insects is a female profession and represents an interesting source of income, although locally seasonal.

Developing insect farming habits may contribute to generation of income, particularly among women, increase yearly availability, and help decrease the impact on the environment from extensive harvesting. More research is needed to assess the nutritional inputs of insects in the Lao diet and to evaluate how far the insect contribution to the Lao diet can be extended.

## Supporting Information

S1 Dataset Dataset on insect’s survey in Laos. (XLS)
